# Artificial intelligence in early onset scoliosis: a scoping review

**DOI:** 10.1007/s43390-025-01208-7

**Published:** 2025-10-19

**Authors:** Chuck Lam, Jennifer Tasong, Halil Bulut, Amy Udall, Tenghis Sukhbaatar, Gary Hoang, Aran Koye, JeeHwan Ahn, Fayez Ghazi, Duncan Loader, Conor T. Boylan, Jwalant S. Mehta, George McKay, Morgan Jones

**Affiliations:** 1 School of Medicine, University of Birmingham, Birmingham, UK; 2 Cerrahpasa School of Medicine, Istanbul University Cerrahpasa, Istanbul, Turkey; 3 School of Medicine, Imperial College London, London, UK; 4 Geisel School of Medicine at Dartmouth, Hanover, USA; 5 Bristol Royal Infirmary, Bristol, UK; 6 Aintree University Hospital, Liverpool, UK; 7 Spine Surgery Unit, Royal Orthopaedic Hospital, Birmingham, UK

**Keywords:** Artificial intelligence, Early onset scoliosis, Machine learning, Spine deformity, Scoping review

## Abstract

**Purpose:**

Early onset scoliosis comprises spinal deformities in children younger than 10, creating challenges in diagnosis, risk assessment, and management. Timely intervention is vital, because untreated deformity can lead to cardiopulmonary compromise. Artificial intelligence and machine learning are reshaping orthopaedic care by improving detection, forecasting progression, and guiding treatment. This scoping review maps current use in this patient population.

**Methods:**

Following PRISMA ScR standards, we systematically searched PubMed, Embase, Web of Science, Cochrane, and Scopus for studies that developed, applied, or validated AI models to diagnose, manage, or predict outcomes in EOS.

**Results:**

After removing duplicates, 352 records were screened, 22 full texts were reviewed, and 11 studies met inclusion criteria. Most investigations (63.6%) employed convolutional neural networks (CNNs) such as Mask R CNN, EfficientNet, and U Net. Ensemble learning with gradient boosting, random forest, and logistic regression (9.1%), Gaussian Naïve Bayes (9.1%), sparse additive machines (9.1%), and unsupervised clustering (9.1%) were also used. Image analysis dominated (72.7%), automating radiographic measurements (Cobb angle, skeletal maturity) and monitoring growing-rod distraction. Predictive models (27.3%) estimated prolonged hospital stay, unplanned reoperation, or postoperative complications. Mean accuracy was 91.2% (range 86.1% to 94.0%). Common limitations were small sample sizes, single-centre data, and limited external validation.

**Conclusion:**

AI shows promise for EOS imaging and risk prediction, yet translation is hindered by methodological heterogeneity and scarce external validation. Future work should adopt standardised reporting, aggregate multicentre datasets, and test models prospectively in large cohorts.

## Introduction

Early onset scoliosis (EOS) refers to a spinal curvature presenting before 10 years of age, encompassing idiopathic, congenital, neuromuscular, and syndromic aetiologies [1]. EOS is clinically challenging due to rapid progression during growth and the risk of thoracic insufficiency syndrome, where a deformed thorax cannot support normal lung development [2–4]. Traditional EOS treatments (casting, bracing, growth-friendly rods) aim to control deformity while facilitating growth. Surgical approaches, however, are associated with high complication rates, [5] frequent unplanned re-operations [6, 7], and prolonged hospitalisations in severe cases [8]. These limitations underscore the need for advanced diagnostic and predictive technologies to support better informed treatment decisions in EOS.

Artificial intelligence (AI) offers such potential. In clinical medicine, AI is most often implemented through machine learning (ML) algorithms that detect relationships in data to classify or predict health states. Deep learning, which uses multilayer neural networks, extends this capability and achieves expert-level performance in diverse spine imaging tasks, for example, automated vertebral segmentation and curvature quantification on radiographs and MRI [9, 10]. ML algorithms can detect patterns across high-dimensional clinical and imaging data that exceed human capability, potentially improving early diagnosis and risk stratification.

In related spinal deformities, AI is already seeing clinical application [11]. In adolescent idiopathic scoliosis, a 2024 scoping review of 40 studies found that most work focuses on automated imaging analysis or prediction of curve progression, but few models undergo external validation [12]. For adult spinal deformity, narrative reviews describe ML tools that classify deformity patterns, anticipate postoperative complications, and personalise surgical planning. Together, these examples highlight AI’s utility across the lifespan of spinal deformity care and provide a foundation for its application in EOS [13].

EOS-specific research is now beginning to mirror these advances. Applications range from computer-vision techniques that measure spinal parameters on radiographs or ultrasound, to predictive models that estimate a patient’s risk of complications or prolonged recovery. By reducing observer variability in measurements and providing prognostic insights, such tools may enhance clinical decision-making. This scoping review aims to map the landscape of AI applications in EOS and summarise current evidence in diagnosis, imaging analysis, treatment planning, and outcome prediction. Ultimately, clarifying the role of AI in the assessment and management of EOS will guide researchers and clinicians in harnessing these technologies to improve care.

## Methods

A systematic scoping review following the PRISMA-ScR (Preferred Reporting Items for Systematic Reviews and Meta-Analyses extension for Scoping Reviews) methodology was conducted. A comprehensive literature search was performed in PubMed, Scopus, CINAHL, Web of Science, and Cochrane from inception to February 2025, using keywords related to “early onset scoliosis,” “artificial intelligence”, “machine learning”, “deep learning”, and “neural networks”. Reference lists of relevant papers were also hand-searched.

### Eligibility criteria

Studies and conference abstracts of any design that applied AI or ML techniques to EOS patients or EOS-related clinical data were included. EOS was defined as scoliosis diagnosed before age 10, irrespective of aetiology. Applications across the spectrum of EOS care (diagnostic imaging, prognostic modelling, treatment guidance, etc.) were also included. Non-English articles, animal or cadaver studies, and papers focusing exclusively on adolescent or adult scoliosis were excluded.

### Study selection

**Fig. 1:**
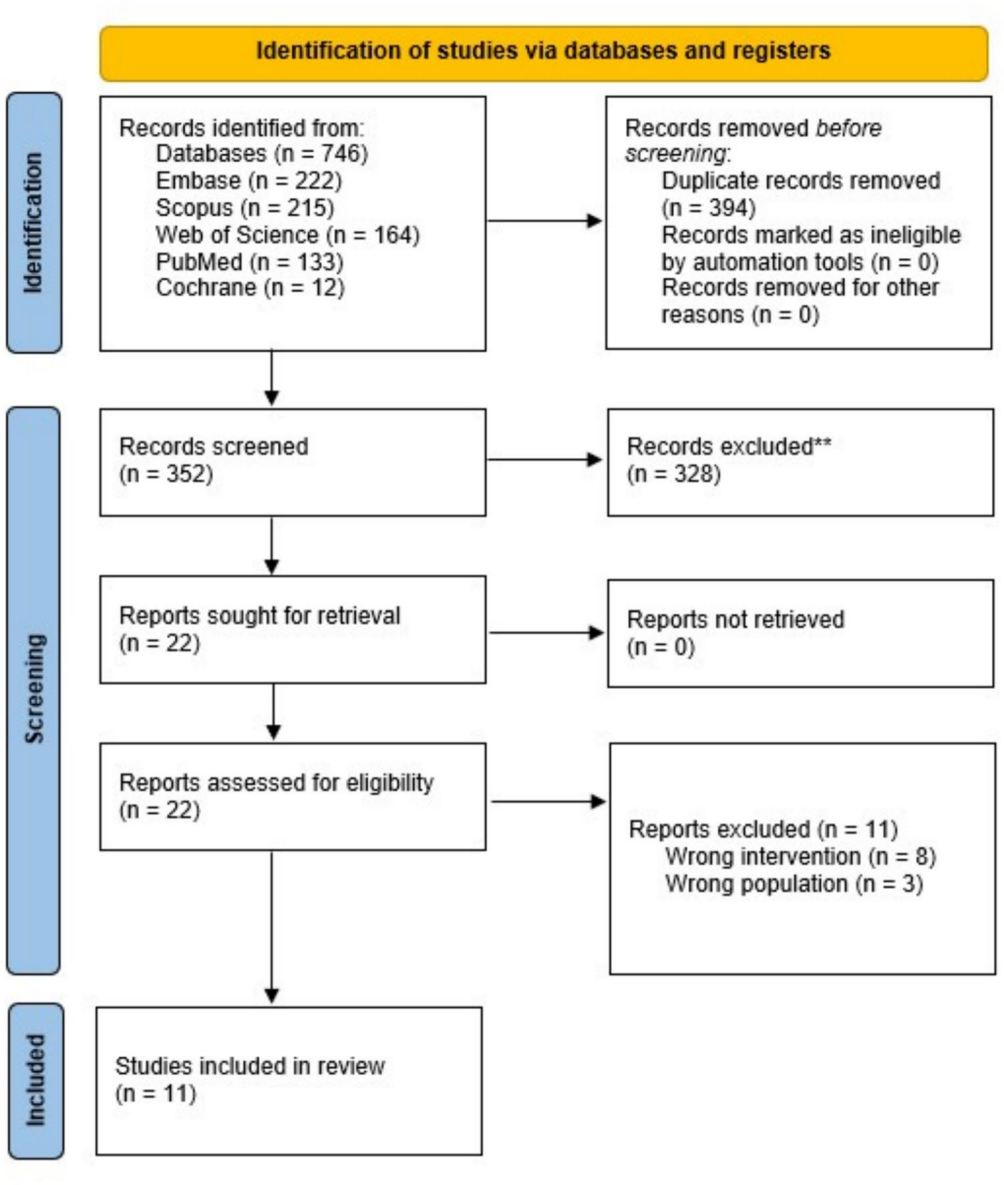
PRISMA Outflow Chart

Search results were exported to Covidence; then, duplicate records were removed using automatic and manual methods. Two reviewers independently screened titles and abstracts. The same reviewers evaluated full texts against the eligibility criteria. Discrepancies were resolved by discussion, and a third reviewer adjudicated when needed. A PRISMA flow diagram was used to document the selection process (Fig. 1).

### Data charting and synthesis

A data charting form to extract key information from each study: publication details, study design, EOS population characteristics, AI/ML techniques used, task or outcome of interest, sample size, and key findings (including performance metrics) was developed. Extracted data were verified by a second reviewer. Given the heterogeneous nature of AI applications, results were synthesised narratively and organised into thematic categories. Illustrative performance metrics (e.g., accuracy, area under the curve, correlation) for each category were documented. Because this is a scoping review, a formal risk-of-bias assessment was not performed, in line with methodological guidance. Instead, study limitations as reported by authors to appraise the evidence were commented on.

## Results

Searching five bibliographic databases yielded 746 records (Embase, 222; Scopus, 215; Web of Science, 164; PubMed, 133; Cochrane CENTRAL, 12). Deduplication removed 394 entries, leaving 352 unique citations for title and abstract screening. Screening excluded 328 citations that did not satisfy the review criteria. The remaining 22 reports were retrieved in full and assessed for eligibility. Eleven full texts were excluded after detailed review, eight because their interventions did not involve AI and three because the study populations were older than ten years at diagnosis. Consequently, 11 studies met all inclusion criteria and were incorporated into the final synthesis (Fig. 1).

### Study characteristics

**Fig. 2:**
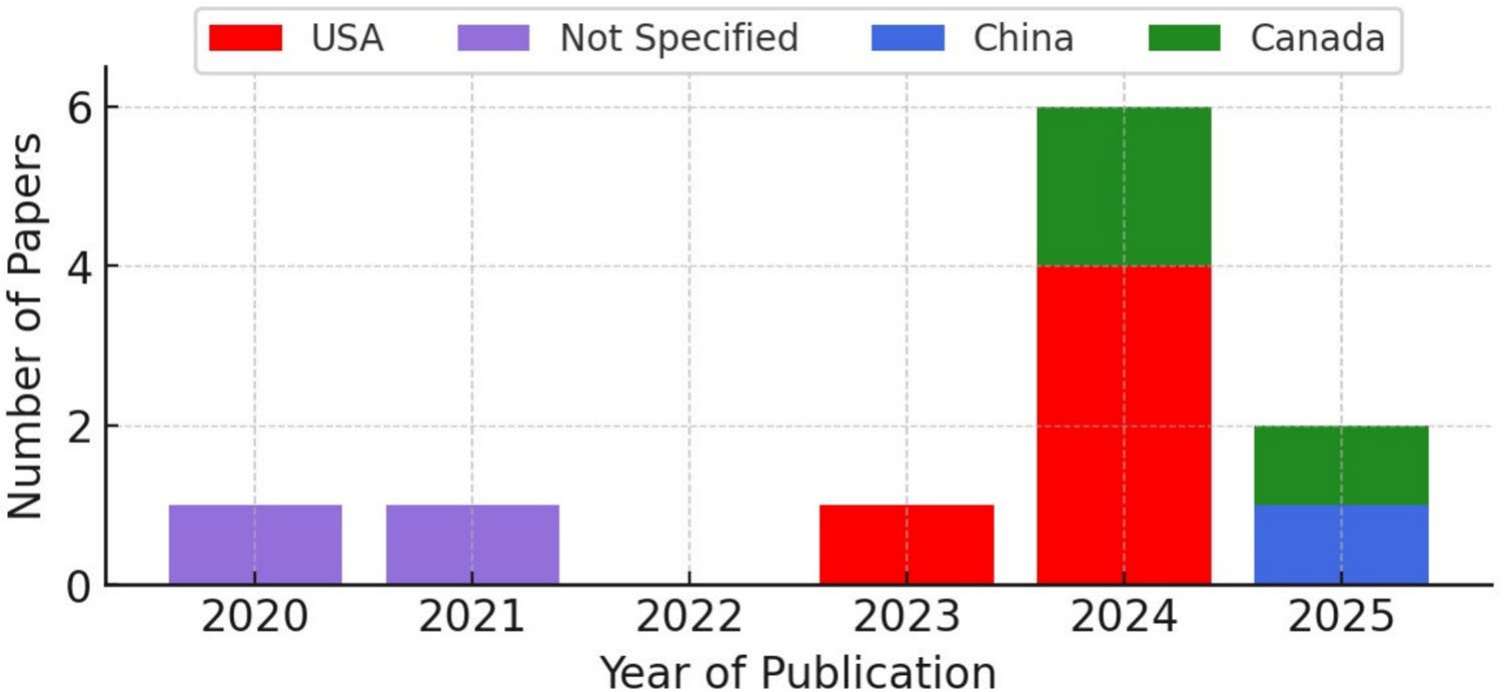
Number of papers by year of publication and country of origin

The included studies [14–24] were published between 2020 and 2025 and encompassed data from over 5,000 paediatric patients. Nearly all studies were conducted in North America, with the USA accounting for the largest proportion (4/11; 36.3%), followed by Canada (2/11; 18.2%) and China (1/11; 9%) (Fig. 2). Table 1 provides an overview of the included studies.

**Table 1:** Summary of Included Studies

Study ID	Application type	Purpose	AI model	Performance metrics	Accuracy/efficacy	Validation
Fields 2024	Predictive	Predict prolonged LOS post-op	Gradient Boosting	AUROC, Accuracy	AUROC: 0.84	Internal fivefold CV
Ha 2020	Diagnostic	Extract skeletal maturity markers	Faster R-CNN + EfficientNet B0	Accuracy, F1	Risser: 92.2%, Sanders: 86.1%	Internal test set
He 2021	Diagnostic	Classify NF1 scoliosis (dystrophic vs non)	Bilateral CNN	Accuracy	90.4%	Internal fivefold CV
Hintz 2024	Imaging	3D spine reconstruction from ultrasound	Attention U-Net	Segmentation Dice Score	Dice = 0.91	External validation
Kabir 2025	Imaging	Measure MCGR length (X-rays)	Detectron2 (Mask RCNN)	Error in mm	Avg error: 1.5 mm	Compared to radiologist
Kabir 2024	Imaging	Measure MCGR length (Ultrasound)	Mask RCNN (Boundary & Rod)	RMSE, Error	RMSE < 2.0 mm	Compared to manual
Lullo 2024	Predictive	Predict return to OR in EOS	Gaussian Naïve Bayes	Accuracy, AUC	AUC: 0.78	70/30 train/test split
Mulford 2024	Diagnostic	Classify paediatric spine X-rays	EfficientNet B6	Top-1 Accuracy	94% overall	Large labelled dataset
Stott 2024	Imaging	Cobb angle measurement	Augmented U-Net	Mean Angle Difference	Mean error: 2.3°	Compared to gold standard
Viraragha van 2023	Diagnostic	EOS subtype clustering	Fuzzy C-means	Silhouette score, Visual similarity	Silhouette score: 0.76	Manual subtype comparison
Han 2025	Predictive	Predict cervical imbalance	Sparse Additive Model	AUC	AUC: 0.81	tenfold CV

### AI modalities

**Fig. 3:**
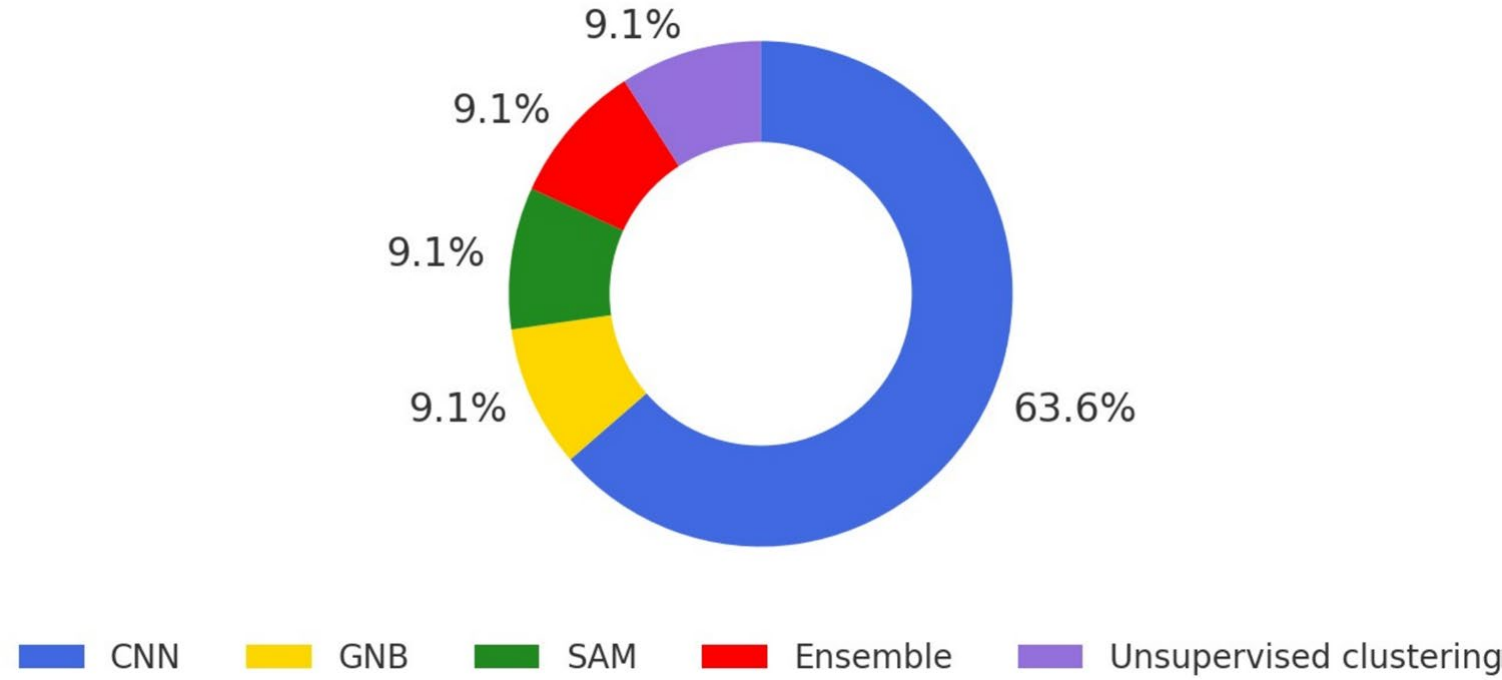
AI Modalities used across studies

The predominant AI technique used across studies was Convolutional Neural Networks (CNNs) (7/11; 63.6%) (Fig. 3). Other models included Gaussian Naïve Bayes (1/11; 9%), Sparse Additive Machine (1/11; 9%), and unsupervised clustering (1/11; 9%). One study used an ensemble of ML techniques (9%), incorporating gradient boosting, random forest, and logistic regression.

The sample datasets used for training and validation exhibited considerable variability in size, ranging from 55 to 10,000 for training sets, and from 15 to 2,008 for testing sets. A majority of studies (9/11; 81.8%) incorporated radiographic imaging parameters as input data, while the remaining studies utilised ultrasound imaging (2/11;18.2%).

Several performance metrics were utilised, with accuracy remaining the most commonly reported (5/11; 45.5 per cent). Of the studies that reported accuracy, values ranged between 86.1% and 94.0%, with a mean accuracy of 91.2%. Other cited metrics included area under the curve (AUC) (3/11; 27.3%), absolute error in millimetres (2/11; 18.2%), root mean square error (2/11; 18.2%), F1-score (1/11; 9.1%), Dice similarity coefficient (1/11; 9.1%), mean angle difference (1/11; 9.1 per cent), silhouette score (1/11; 9.1%) and visual similarity score (1/11; 9.1%).

### Application of AI models

**Fig. 4:**
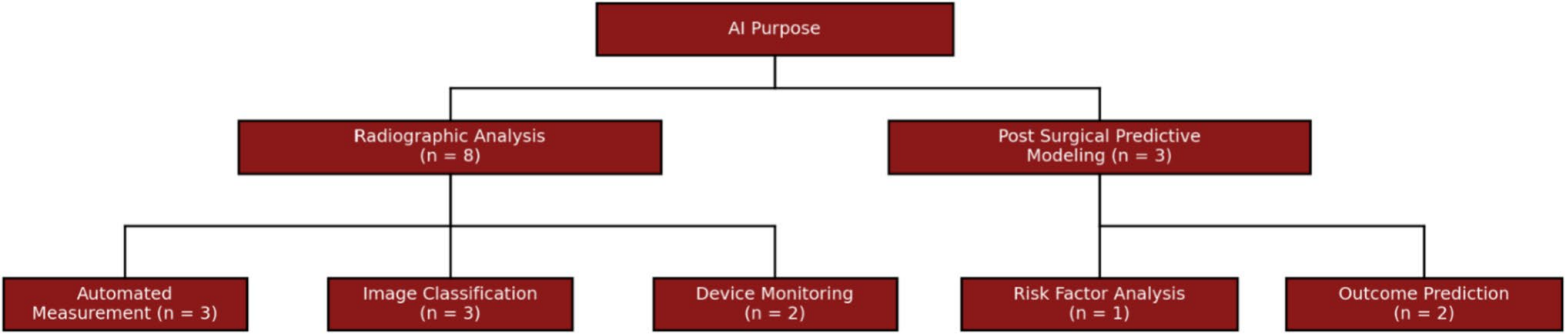
Applications of AI categorised by main themes and subthemes

The application of AI models within studies fell into two broad themes: Radiographic Analysis and Measurement, and Predictive Modelling for Surgical Outcomes. Figure 4 presents the breakdown of AI applications into major themes and their associated subcategories. Below, the main findings within each thematic category are described.

### Radiographic analysis and measurement

A total of eight models (8/11 72.7%) were applied to radiographic imaging and analysis. Of these, three main sub-applications emerged: (1) automated measurement, (2) image classification and categorisation, and (3) therapeutic device monitoring. These studies are summarised in Table 2.

**Table 2:** Studies looking at radiographic analysis and measurement

Study ID	AI model	Purpose
Hintz 2024	Attention U-Net	Segment bone, reconstruct 3D model, measure scoliosis
Kabir 2025	Detectron2-based Mask R-CNN	Measure MCGR length on radiographs
Kabir 2024	Mask R-CNN (Boundary & Rod models)	Extract and measure MCGR length from ultrasound
Stott 2024	Augmented U-Net	Automated Cobb angle measurement for scoliosis
Ha 2020	Faster R-CNN Inception V2 + EfficientNet B0	Extract multiple skeletal maturity classifications
He 2021	Bilateral CNN	Classify dystrophic vs. nondystrophic scoliosis
Mulford 2024	EfficientNet B6	Classify radiographs by surgical stage, brace status, and implant type
Viraraghavan 2023	Fuzzy C-means clustering	Unsupervised classification of EOS patients into subgroups

Three studies (3/8; 37.5%) demonstrated the potential of AI to automatically extract metrics such as Cobb angles and spinal curvature from radiographs. Consequently, reducing the excess radiation exposure and financial costs incurred by additional radiographs. Hintz et al. (2024) reported the reliability of an ML model to measure the transverse process angle from ultrasound images. This radiation-free approach attained an average angular variation of ± 1.3° (SD = 1.1), in comparison with radiographic imaging, evidencing its ability to accurately assess paediatric scoliosis.

A number of studies (3/8; 37.5%) also explored the classification of spinal images into treatment phases and subtypes. He et al. (2021) developed a deep learning CNN to automatically differentiate radiographs into pre-operative, post-operative, in-brace and out-brace categories. A near-perfect performance was achieved, exhibiting an accuracy of 1.00 in both AP/PA and lateral radiographs and precision values ranging from 0.98–1.00 and 0.91–1.00 in AP/PA and lateral radiographs, respectively.

The remaining two studies (2/8; 25.0%) employed AI for monitoring therapeutic devices (25.0%), particularly focusing on growing rod lengths. One study by Kabir et al. (2024) applied a Mask R-CNN-based boundary model to analyse the ultrasound images of 23 paediatric patients with magnetically controlled growing rods (MCGRs). The model demonstrated a high level of reliability and highlighted the potential to increase efficiency and reduce costs when monitoring MCGRs. An average precision of 88.5% was reported in the boundary model, alongside a 1.2 mm (SD = 1.46) MAD between expert and AI measurements.

### Predictive modelling for surgical outcomes

Three studies (3/11; 27.2%) investigated predictive modelling for post-operative outcomes. Among these, one study (1/3; 33.3%) focused specifically on analysing risk factors for cervical sagittal imbalance following growing rod surgery. The models identified significant risk factors such as increases in cervical lordosis and post-operative proximal junctional kyphosis.

Similarly, two studies (2/3; 66.6%) addressed the prediction of post-operative outcomes, including prolonged hospital length of stay (LOS) and unplanned returns to the operating room (UPROR). These models showed promising capabilities in effectively identifying patients at risk of an extended hospital LOS and UPROR. A study by Fields et al. (2024) reported an AUC of 0.741 in a gradient boosting model designed to predict prolonged hospital LOS from a testing set of 318 patients. In a complementary investigation, Lullo et al. (2024) achieved an AUC of 0.79 with a model developed to forecast patients requiring a UPROR. The favourable outcomes observed from these studies highlight the potential for AI to enhance surgical planning and aid informed resource allocation. These studies are summarised in Table 3.

**Table 3:** Studies Focusing on Predictive Analytics

Study ID	AI model	Purpose
Fields 2024	Gradient Boosting (best performer among ensemble classifiers)	Predicting prolonged hospital length of stay (> 5 days)
Han 2025	Sparse Additive Machine (SAM)	Predict risk factors for cervical sagittal imbalance after growing rod surgery
Lullo 2024	Gaussian Naïve Bayes	Predict need for unplanned return to OR

## Discussion

This scoping review focused on the use of AI in the care of EOS. The small body of the literature identified reveals that research in this area is in its early stages. Most studies concentrate on image-based tasks; two-thirds of models analysed radiographic or ultrasound data to automate measurements or classify the phase of treatment. A smaller subset developed predictive models for surgical outcomes, while a few employed unsupervised clustering to identify patient subgroups. Overall, reported accuracies were generally high on internal test sets (mean 91.2%, range 86.1% to 94.0%), but sample sizes were small and there was minimal external validation, limiting generalisability.

### Interpreting algorithm performance

Across the studies we reviewed, high accuracy usually reflected the type of problem and the data used, not a single best algorithm, so pooling results across studies was not appropriate. Convolutional neural networks worked best for image tasks on radiographs, because they capture local detail and patterns at different scales, and several systems now produce clear overlays that clinicians can check. Spine ultrasound benefited from U Net style models that include attention or prior information, these help the model link distant image regions and reduce noise, which improves segmentation and curvature visualisation. For prediction tasks that use clinical variables, for example, length of stay or unplanned return to the operating room in paediatric deformity, tree-based methods and gradient boosting often performed well because they handle mixed data types and smaller sample sizes with built in regularisation, but performance changed when outcomes were defined differently, or labels were noisy. In short, models did best when the metric matched the clinical goal and labels were reliable, for example, absolute error for angle measurement.

### AI for imaging analysis and device monitoring

The dominant application of AI in EOS was automated image analysis. Mask R-CNN and EfficientNet-based models successfully segmented bones and measured parameters such as Cobb angle, thoracic kyphosis and axial vertebral rotation from radiographs and 3D ultrasound images. One attention-based U-Net reconstructed three-dimensional spinal volume from motion-tracked ultrasound, producing clinically acceptable measurement variance (mean absolute differences under 1.3° for transverse process angle) and demonstrating the feasibility of automated radiation-free monitoring [14]. Another model classified radiographs by surgical stage, brace status and implant type, achieving near-perfect accuracy and precision values up to 1.00 for differentiating pre-operative and post-operative images [20]. The ability of these CNNs to rapidly and consistently generate quantitative measurements could reduce inter-observer variability and decrease radiation exposure from repeated radiographs [25, 26], and lower costs by reducing manual measurement time.

One study applied AI to monitor therapeutic devices. A Detectron2-based Mask R-CNN accurately measured the length of magnetically controlled growing rods on radiographs [16], while a boundary model and rod model combined Mask R-CNN architectures to measure rod length using ultrasound, reporting an average error under 2 mm. Such tools could facilitate non-invasive, real-time tracking of rod expansion during the distraction phase of treatment, potentially obviating additional radiographs and shortening clinic visits, which can reduce direct imaging costs and staff time while preserving clinic throughput.

### Predictive modelling for post-operative outcomes

Three studies used machine learning to predict clinical outcomes following EOS surgery. Sparse additive machine models analysed radiographic and clinical data to identify risk factors for cervical sagittal imbalance following growing rod surgery [21]. They found that postoperative proximal junctional kyphosis (PJK), greater improvement of kyphosis, and larger increases in T1-slope were the strongest predictors of sagittal malalignment. Gradient boosting and Gaussian naïve Bayes classifiers predicted prolonged hospital length of stay and unplanned returns to the operating room, achieving area under the curve values of 0.74–0.79. These preliminary findings suggest ML can uncover complex associations among surgical parameters and may inform perioperative risk stratification. However, the models were trained on retrospective data from single institutions and were not externally validated; thus, their clinical utility remains uncertain.

### Clinical integration, validation, and standards for future synthesis

Moving these tools into practice needs evidence that goes beyond internal accuracy. Models should be tested on independent cohorts, with calibration shown, for example, calibration plots and Brier score, and, when needed, updated to local data. Clinical value should be demonstrated using decision curve analysis alongside AUC or similar discrimination metrics. To make results comparable across studies, future work should agree on outcome definitions and a small, task matched set of metrics, for example, Dice and absolute error for measurement tasks, AUC with calibration for prediction, plus confusion matrices or calibration plots. At present, heterogeneity in inputs, outcome thresholds, and reported metrics limits the feasibility of formal comparisons or meta-analyses and makes cross study pooling inappropriate, standardisation is required before random effects synthesis or summary ROC approaches will be meaningful. Governance and reporting should follow current best practices and align with regulatory guidance on statistical validation and monitoring for dataset shift. Finally, deployment should fit routine workflow, integrate with PACS and electronic health records, provide editable overlays and confidence information, and return results within normal reporting times.

### Limitations of current research and future directions

Despite promising results, the EOS AI literature is characterised by small sample sizes, single-centre data and heterogeneous methodologies. Most models were developed using fewer than 400 cases, and many studies provided limited detail on data preprocessing, algorithm version or validation strategy. None of the included studies used longitudinal or multi-centre datasets, limiting generalisability. Moreover, no study combined imaging with clinical or biomechanical data, even though multimodal fusion has improved predictions in other medical fields. The prevalence of black-box models also raises concerns about interpretability, as clinicians cannot easily understand how predictions are derived. Future research should employ explainable models or augment deep networks with interpretability techniques to enhance clinician trust and patient safety.

To advance AI in EOS, several steps are necessary. Larger, multi-centre cohorts that include imaging, clinical variables and patient-reported outcomes are essential for training robust models and enabling external validation. Researchers should explore multimodal approaches that integrate radiographs, ultrasound, clinical notes, and biomechanical measures to capture the multifaceted nature of EOS. Rigorous reporting standards such as the SPIRIT-AI, TRIPOD + AI, and CONSORT-AI standards should be adopted to ensure transparency and reproducibility [27–29]. Prospective studies and randomised trials are needed to demonstrate whether AI tools improve patient outcomes or reduce complications in real-world settings. Ethical considerations related to data privacy, bias, and equitable performance across diverse populations must also guide future development. Through interdisciplinary collaboration and adherence to best practices, AI has the potential to become a valuable adjunct in the management of EOS.

Adoption patterns in EOS will reflect broader changes across healthcare specialties that are integrating AI. As imaging automation, risk prediction, and decision support tools gain traction in other areas, similar capabilities are likely to move into EOS workflows. As a specialty, planning for this trajectory is advisable, for example, by building data infrastructure and establishing evaluation and governance pathways that accommodate AI-enabled tools.

## Conclusion

Early work on artificial intelligence for early onset scoliosis is promising but preliminary. Most of the eleven eligible studies concentrate on image-based tasks, where convolutional neural networks reliably automate Cobb-angle and device-monitoring measurements, while a smaller group of models predicts outcomes such as prolonged hospital stay or unplanned re-operation. Reported internal accuracies are high, yet nearly all studies use small, single-centre datasets and lack external validation or clear implementation pathways, limiting clinical generalisability. To move from proof-of-concept to practice, future research must adopt larger multicentre cohorts, follow AI-specific reporting standards, and incorporate explainable and multimodal approaches that align with real-world decision-making in the management of early onset scoliosis.

## Data Availability

All data is available upon reasonable request to the corresponding author.
